# Changes in Fertility at the Population Level in the Era of ART in Rural Malawi

**DOI:** 10.1097/QAI.0000000000001395

**Published:** 2017-05-01

**Authors:** Estelle McLean, Alison Price, Menard Chihana, Ndoliwe Kayuni, Milly Marston, Olivier Koole, Basia Zaba, Amelia Crampin, the ALPHA Network

**Affiliations:** * Faculty of Epidemiology and Population Health, London School of Hygiene and Tropical Medicine, London, United Kingdom; and; † Malawi Epidemiology and Intervention Research Unit, Lilongwe, Malawi.

**Keywords:** HIV, ART, Africa, fertility, Malawi

## Abstract

**Introduction:**

HIV reduces fertility through biological and social pathways, and antiretroviral treatment (ART) can ameliorate these effects. In northern Malawi, ART has been available since 2007 and lifelong ART is offered to all pregnant or breastfeeding HIV-positive women.

**Methods:**

Using data from the Karonga Health and Demographic Surveillance Site in Malawi from 2005 to 2014, we used total and age-specific fertility rates and Cox regression to assess associations between HIV and ART use and fertility. We also assessed temporal trends in in utero and breastfeeding HIV and ART exposure among live births.

**Results:**

From 2005 to 2014, there were 13,583 live births during approximately 78,000 person years of follow-up of women aged 15–49 years. The total fertility rate in HIV-negative women decreased from 6.1 [95% confidence interval (CI): 5.5 to 6.8] in 2005–2006 to 5.1 (4.8–5.5) in 2011–2014. In HIV-positive women, the total fertility rate was more stable, although lower, at 4.4 (3.2–6.1) in 2011–2014. In 2011–2014, compared with HIV-negative women, the adjusted (age, marital status, and education) hazard ratio was 0.7 (95% CI: 0.6 to 0.9) and 0.8 (95% CI: 0.6 to 1.0) for women on ART for at least 9 months and not (yet) on ART, respectively. The crude fertility rate increased with duration on ART up to 3 years before declining. The proportion of HIV-exposed infants decreased, but the proportion of ART-exposed infants increased from 2.4% in 2007–2010 to 3.5% in 2011–2014.

**Conclusions:**

Fertility rates in HIV-positive women are stable in the context of generally decreasing fertility. Despite a decrease in HIV-exposed infants, there has been an increase in ART-exposed infants.

## INTRODUCTION

Fertility in many countries of sub-Saharan Africa (SSA) is falling,^1^ although the rate of decline has been slower than most other world regions, and there is some evidence to suggest that fertility decline has plateaued in some countries.^2^ Fertility is influenced by childbearing desires, contraceptive use, and maternal health. In SSA, increases in women's education and employment opportunities, access to contraception, and changes in societal and individual opinions regarding delaying childbearing in unstable or uncertain periods are thought to have influenced fertility trends.^3^ In many SSA countries, the HIV epidemic has had negative impacts on health and family stability with consequences for fertility.^4–6^

The effect of HIV on fertility is complex. HIV and fertility are positively associated in adolescents, as women who have unprotected sex have higher risk of both HIV acquisition and pregnancy.^7^ However, older HIV-positive women have lower fertility rates than HIV-negative women.^4,7^ HIV infection has been shown through biological pathways to reduce the ability to conceive and bear a live child.^8–12^ Social and behavioral factors are also related to lower fertility in HIV-positive women because of altered childbearing desires^11,13–16^ and likelihood of being in a relationship.^17^ In Malawi and other high HIV-prevalence countries, widespread availability of antiretroviral treatment (ART) has removed many of the biological barriers to fertility by improving health and survival of women and their partners,^18,19^ which has been shown to increase people's desire for children,^20,21^ although this may not correlate with actual fertility.^22^ There is, however, still uncertainty surrounding the impact of increased access to ART on fertility in HIV-positive women.^23^

Malawi has adopted a public health approach^24^ to its HIV epidemic with sector-wide policies to maximize ART uptake in HIV-positive people, including Option B+^25^: from July 2011, all pregnant and breastfeeding women were eligible for lifelong ART regardless of immunological status, and in 2016, universal ART eligibility was introduced so all HIV-positive people should be offered ART at the time of testing.^26^ Before Option B+, prevention of mother-to-child transmission programmes involved HIV testing for pregnant women and short courses of ART, taken until completion of breastfeeding.^27^ Since 2011, the guidelines in Malawi have promoted provider-initiated family planning but specifically stated that “Health workers should not actively discourage pregnancy” in HIV-positive women.^28^

Studying trends in fertility is important, as it enables long-term planning for services required for pregnant women and their children at various stages in their lives. In Malawi, infants born to HIV-positive mothers should be monitored until aged 24 months, which presents a burden to an already stretched health service, and retention in this programme is not always optimal.^29^ Reducing the number of children born with HIV would reduce the burden on the health system once they were older, but there is little evidence regarding the long-term effects of exposure to ART in utero or through breastfeeding, especially in rural African contexts.^30^ If the expansion of the ART programme is accompanied by increasing fertility in women on ART, there will be a concomitant increase in the number of infants requiring monitoring and testing for HIV and an increasing number of ART-exposed children whose future health needs are currently uncertain.

In this study, we use data from a rural longitudinal cohort in northern Malawi to explore temporal fertility trends and the association of HIV infection and ART availability with fertility. We also describe temporal trends in HIV and ART exposure in infants.

## METHODS

### Population and Data

#### Population

The Karonga Health and Demographic Surveillance Site (HDSS), established in 2002, captures information on births, deaths, and in- and out-migrations in a population of approximately 39,000 people (including 9000 women aged 15–49) living in a 150 km^2^ rural area of northern Malawi.^31^ The HDSS has also collected socioeconomic information, including highest attained education level and marital status, from annual surveys since 2007. In 2007–2008, the adult HIV prevalence was 6.3% in males and 8.5% in females, and estimated ART coverage was 48% in males and 65% in females in 2008–2010.^32^

#### HIV Test Data

Within the HDSS, population-level HIV testing and counseling in adults 15 years and older were done in multiple serosurveys, starting with randomly selected clusters in 2005–2006 and followed by 4 successive HIV serosurveys across the whole population, from 2007 to 2011. Interviewers asked about previous HIV testing (available from several providers in the area), including the timing and result of the most recent test (positive, negative or unknown). Each serosurvey used a standardized rapid test protocol; results were immediately available to the participant^33^ and the vast majority (>90%) of people consented to be informed of their result. Within the HDSS, HIV testing (with disclosure to participants) has also been offered in some smaller research studies conducted from 1988 onward. We included these HIV test data in this analysis, if the study was population representative and not limited to select groups (eg, clinically symptomatic people).

For our analysis, we assigned HIV-positive status from 1 year before their first positive test date (or from the midpoint between the last negative test and the first positive, if that interval was 1 year or shorter). To be comparable with the HIV-positive women, we assigned women as HIV-negative from 1 year before their first negative test date (self-report or study), and up to 4 years after their last negative test date (because of relatively low incidence rates in the area and length of time since the last serosurvey) unless they had a subsequent positive test during this time. All other time was classified as unknown. Infants were categorized as HIV exposed if their mothers were identified as HIV positive at any time up to 1 year after their birth (assuming that they would have been breastfed for at least 1 year).

#### ART Use Data

ART was available at the main hospital (which is 70 km from the HDSS area and largely inaccessible to the HDSS population) from June 2005, at the rural hospital within the HDSS area from September 2006, and at smaller clinics in the area from October 2010. Data on uptake of HIV services were collected through links with local clinics: by identifying and tracking cohorts of consenting people initiated on ART and capturing current and previous ART usage.^34^ We assigned women as not (yet) on ART from 1 year before their first positive test (ie, from the point we assigned them as HIV positive) up to their first ART start date (self-reported or clinic) or up to 2 years after the last date they are known to have never taken ART. We assigned women as on ART from their first ART start date (self-reported or clinic) up to their last ART stop date (self-reported or clinic) or up to 3 months after the last date they are known to still be taking ART (assuming they took their final prescription). Infants were categorized as ART exposed if their mothers were on ART at any time up to 1 year after their birth (assuming that they would have been breastfed for at least 1 year).

### Statistical Methods

We conducted a longitudinal analysis, including person years for the period of follow-up for the entire population of women aged 15–49 and all live births (with multiple births contributing separately) born to these women between 2005 and 2014. We examined fertility trends over 3 periods of ART availability: 2005–2006 (no/little ART in the HDSS area), 2007–2010 (ART roll-out), and 2011–2014 (ART widely available) in the following ways:

- We calculated age-specific fertility rates by HIV/ART status. We then used these age-specific rates to calculate the total fertility rate (TFR): a summary measure of the average number of children that would be born per woman if they were to complete childbearing years experiencing the current age-specific rates. We also used Cox proportional hazards regression models to compare fertility rates in HIV-positive women on ART or not (yet) on ART with those of HIV-negative women, with adjustment for age (in 5-year age bands), marital status (never married, married, divorced, and widowed), and educational level (none, primary standards 1–3, primary standards 4–7, primary completed, 2 years of secondary, and 4 years of secondary). Option B+ has resulted in relatively healthy women initiating ART because of pregnancy; to avoid this artificially raising the apparent fertility of women on ART, we only included women who had been on ART for at least 9 months (excluding 102 births where the mother started ART during pregnancy and 486.3 person years). Subsequent births to Option B+ women were included.
- We calculated crude and age-specific fertility rates by number of years on ART (excluding less than 1 year because of the above described issue of women starting ART in pregnancy and combining 4 years and above because of the small number of births in younger women).
- We calculated the number and proportion of infants who were HIV and ART exposed in the 3 periods and the proportion exposed to ART from the first trimester of pregnancy until at least delivery (calculated by estimating the conception date as 9 months before the birth date).

## RESULTS

From 2005 to 2014, there were 13,583 live births during approximately 78,000 person years of follow-up of women aged 15–49. Overall, the TFR was 5.2 [95% confidence interval (CI): 4.9 to 5.4], declining from 6.0 (5.4–6.6) in 2005–2006 to 4.6 (4.3–4.9) in 2011–2014 (Table 1). The highest crude fertility rates were in the 20–24 year age group [270.7 per 1000 person years (262.5–279.2)], in married women [226.2 per 1000 (221.8–230.7)], and in women with 4–7 years of education (excluding unknown education category) [182.6 per 1000 (177.6–187.7)] (Table 1). There were 9391 births to HIV-negative women and 626 births to HIV-positive women (158 on ART for at least 9 months, 294 not on ART, 102 started ART during pregnancy, and 72 unknown) (Table 1). Of the 1294 women reported to be HIV positive during the analysis, 738 (57.0%) had their first HIV-positive test date derived from clinic or HIV test data and 556 (43.0%) from self-reported data. Of the 745 women reported to be on ART at any point during the analysis, 380 (51.0%) had the first ART initiation date derived from clinic data and 365 (49.0%) from self-reported data.

**TABLE 1.**
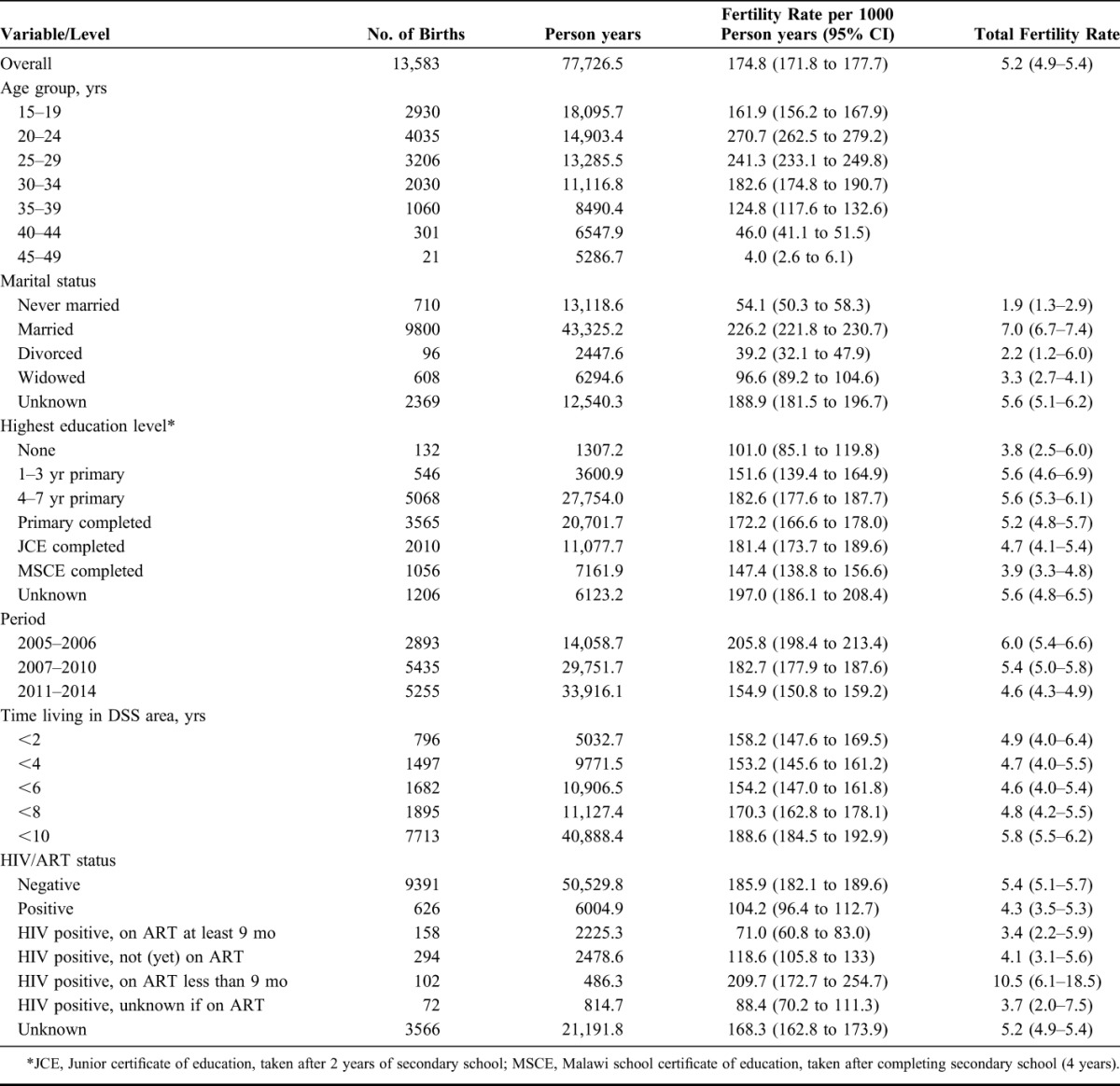
Number of Births, Person Years, Crude Fertility Rate, and Total Fertility Rate by Demographic and HIV/ART Variables, 2005–2014

### Fertility Rates in HIV-positive vs. HIV-negative Women

From 2005–2006 to 2011–2014, there was a downward trend in the TFR of HIV-negative women [6.1 (4.6–8.1) to 5.1 (4.8–5.5)]. In HIV-positive women, there was an increase from 3.7 (1.7–8.4) in 2005–2006 to 4.3 (3.2–6.0) in 2007–2010. However, it remained stable up to 2011–2014 when it was 4.4 (3.2–6.1) (Table 2). The TFR in women with unknown HIV status decreased more sharply than in the HIV-negative women, from 6.1 (5.5–6.8) to 2.8 (2.1–4.0); this was due to a fall in the number of births born to women with unknown HIV status (669 in 2007–2010 vs. 371 in 2010–2014), which was not reflected in a decrease in the number of person years (4300.9 in 2007–2010 vs. 4919.6 in 2010–2014).

**TABLE 2.**
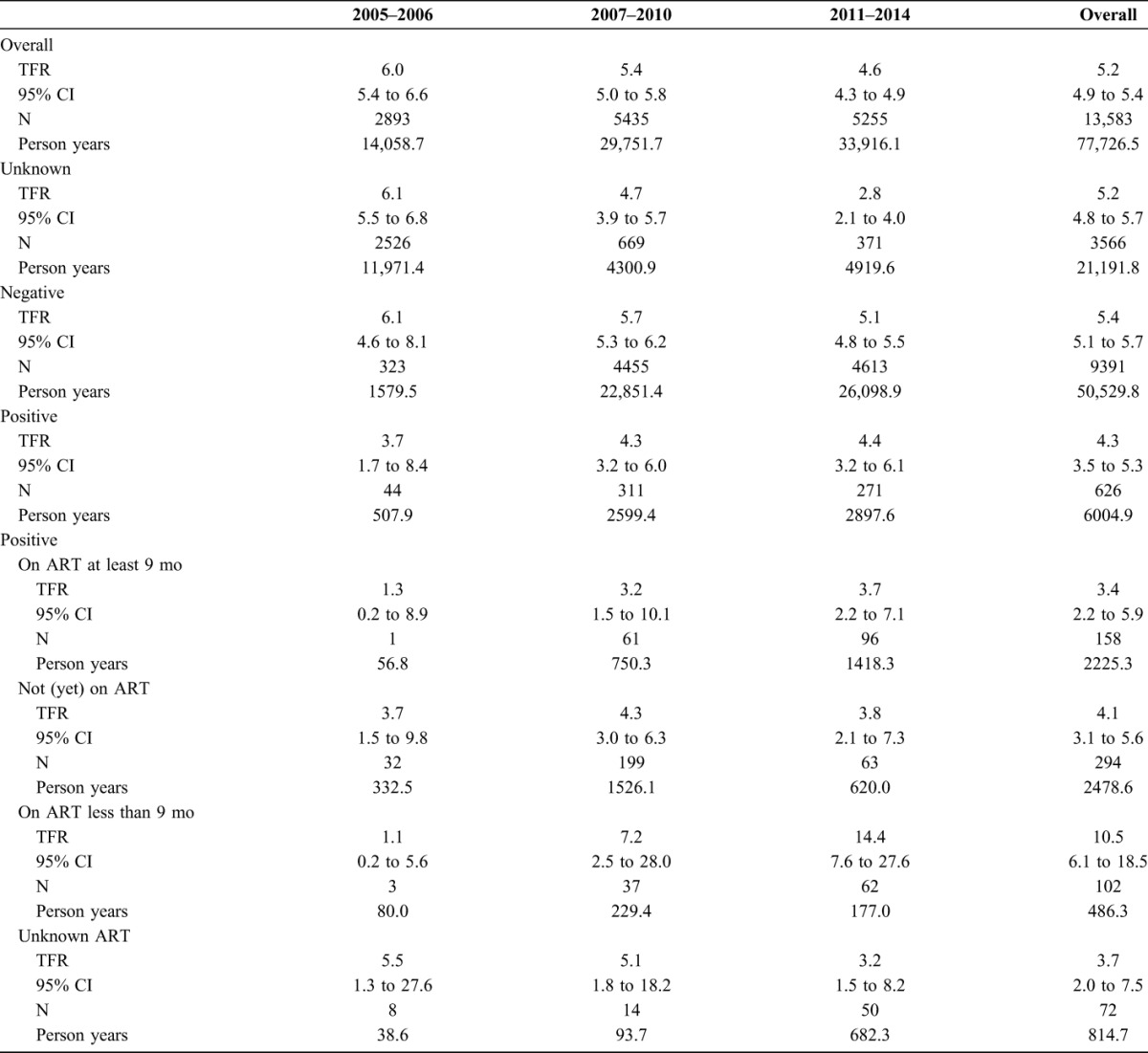
Total Fertility Rate (and Number of Births) by HIV/ART Status and Year

### Fertility Rates in HIV-positive Women on ART vs. HIV-positive Women Not on ART

**FIGURE 1.**
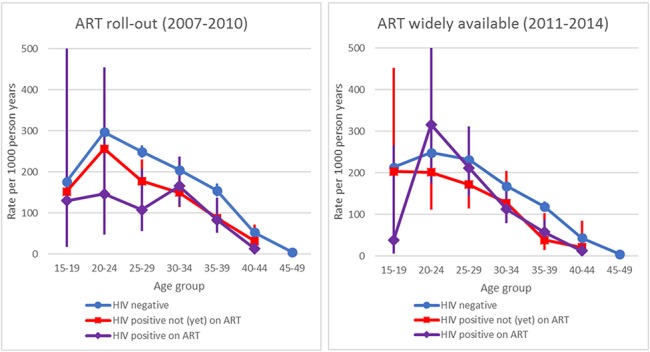
Age-specific fertility rates by HIV/ART status and era of ART availability. NB. Vertical lines show confidence limits around each estimate (capped at 500).

The pattern of age-specific fertility rates in women on ART for at least 9 months changed from 2007–2010 to 2011–2014. In the earlier period, rates in younger women were lower than those of women not on ART, whereas in the later period, they were higher and more similar to those of HIV-negative women. However, CIs were wide: the fertility rate in the 20–24 year group on ART at least 9 months was 146.5 per 1000 (47.3–454.3) in 2007–2010 and 316.0 per 1000 (175.0–570.6) in 2011–2014. Rates in older women in both periods were similar in women on and not (yet) on ART (Fig. 1).

The TFR in HIV-positive women on ART for at least 9 months in 2011–2014 was 3.7 (2.2–7.1), which was similar to that of women not on ART [3.8 (2.1–7.3)] (Table 2). Compared with HIV-negative women, HIV-positive women experienced lower adjusted hazards for a live birth throughout follow-up, and there was little evidence of a difference between women on ART for at least 9 months and not (yet) on ART: in 2011–2014, the adjusted hazard ratios were 0.7 (0.6–0.9) and 0.8 (0.6–1.0), respectively (Table 3). The TFR in women who started ART during pregnancy was high and increased over time. The rates for women with unknown ART status were higher than those known not to be on ART; however, the numbers of births and person years in both these categories were small (Table 2).

**TABLE 3.**
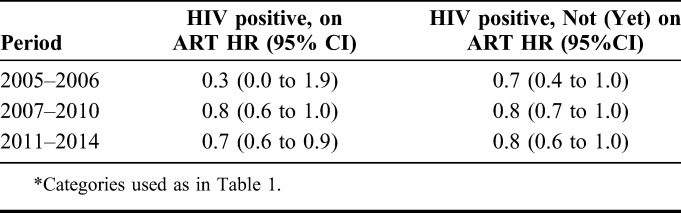
Hazard Ratio (HR) of Live Birth Event Controlling for Age, Marital Status, and Education Level* in HIV-positive Women Compared With HIV-negative Women by ART Usage and Year

### Fertility Rates in HIV-positive Women by Years on ART

Crude fertility rates increased with years since ART initiation, from 65.4 per 1000 (46.5–91.9) in women on ART for 1 year, to a peak of 94.0 per 1000 (95% CI: 66.8 to 132.3) in women who had been on ART for 3 years. The rate in women on ART for 4 or more years was lower at 64.8 per 1000 (49.4–85.0). This pattern was observed in the 20–29 year age group, and, at lower levels, in the 30–49 year group (Table 4).

**TABLE 4.**
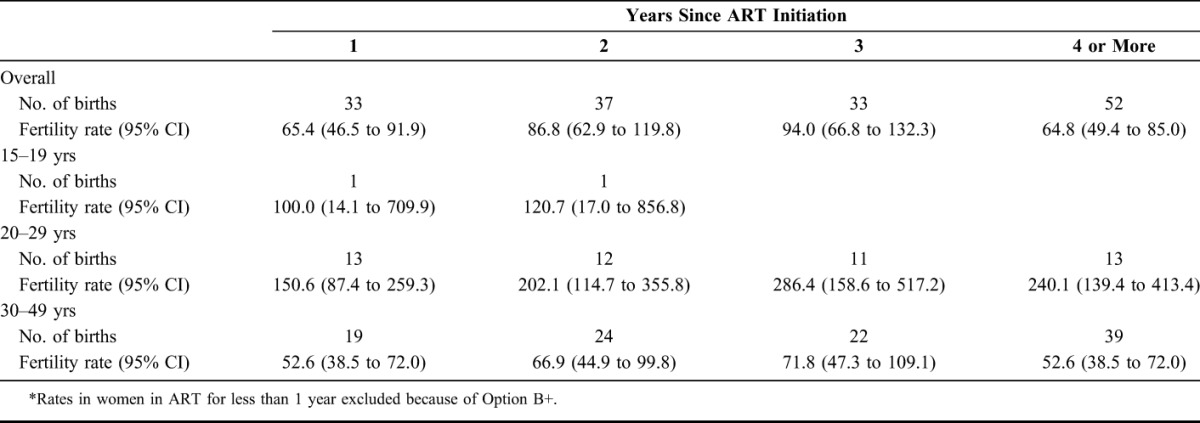
Fertility Rates in HIV-positive Women on ART by Years Since ART Initiation*

### Exposure to ART in Utero and Breastfeeding

The proportion of infants who were HIV exposed decreased slightly from 5.8% (313/5435) in 2007–2010 to 5.2% (271/5255) in 2011–2014. The proportion exposed to ART increased, however, from 2.4% (83/5435) to 3.5% (185/5255). In 2011–2014, 2.5% (n = 134) of all live births were reported to have been exposed to ART from the first trimester of pregnancy up to delivery.

## DISCUSSION

Our findings from rural Malawi show that from 2005 to 2014, during increasing availability of ART, the TFR declined overall and in HIV-negative women, while remaining stable and lower in HIV-positive women. There was a suggestion of an increase in fertility levels in younger women on ART, and fertility levels increased with time on ART (up to peak at 3 years since initiation); however, there was little overall difference in fertility in women on ART for at least 9 months and women not (yet) on ART. Nonetheless, despite lack of evidence for increasing fertility in HIV-positive women at the population level, the proportion of infants exposed to ART has increased.

The overall decrease in fertility in our HDSS is consistent with the level of fertility decline shown in the 2010 Malawian Demographic and Health Survey, which reported a TFR of 5.7 for the northern region, which compares well to our figure of 5.4 in 2007–2010.^35^ Our findings of increased fertility rates in HIV-positive women, although lower than HIV-negative women during the era of widespread ART availability, are consistent with those of a systematic review of recently published studies.^23^ Evidence for an effect of duration of ART on fertility is inconsistent: some studies report no difference while others, including ours, report increasing fertility rates with increased duration of ART.^23^

The lower fertility rates in younger women on ART in the early period of ART availability in our data may be because ART was initially provided to the sickest (and presumably least fertile) individuals, with treatment extending to individuals at an earlier stage of disease over time. Recently, movement of HIV-positive women onto ART at an earlier stage of disease, may explain the similar fertility levels in women on ART and those not (yet) on ART (who are yet to experience fertility effects of poor health). Prolonged use of ART may itself have biological effects on fertility which may explain the lower fertility levels of HIV-positive women on ART compared with HIV-negative women, and that even in younger women, fertility rates in women on ART for 4 or more years are lower than those in women on ART for 3 years. There is evidence to suggest that some antiretroviral drugs have an adverse effect on gametes,^8^ although the effect of ART on male fertility is unclear.^36,37^ Early evidence on the impact of ART on birth outcomes is inconsistent, with some studies showing no effect,^38^ whereas others report increased preterm births.^39^

Fertility dynamics are also affected by childbearing desires. Although evidence suggests that HIV-positive women are less likely to want more children than HIV-negative women,^15^ a study of 9 SSA countries found no consistent pattern in changing fertility desires during increasing coverage of ART.^16^ Although guidelines recommend that HIV-positive women are not actively discouraged from becoming pregnant,^40^ HIV-positive women in Malawi reported no or discouraging discussions about childbearing with health care workers^41^; this was confirmed by health care workers in the same area who had mixed attitudes around childbearing in HIV-positive women, with several actively discouraging women.^42^ Community stigma against childbearing in HIV-positive women may also explain why fertility levels in HIV-positive women are not the same as those in HIV-negative women.

Excluding women who start ART during pregnancy was necessary to examine the effect of ART on fertility, as many of these women would have only started ART because of the pregnancy while healthy and the treatment would have had no effect on the conception and live birth of the child (so including them would inflate the observed effect of ART). Nonetheless, it is also plausible that ART could have reduced the chance of a miscarriage or, greater knowledge of ART availability with the introduction of Option B+ may have encouraged HIV-positive women/couples to bear a child when otherwise they might not have; therefore, it is possible that we have underestimated the effect of ART on fertility in the population.

We found a reduction in the number of HIV-exposed infants in recent years but, mainly due to the Option B+ policy, an increasing number of infants exposed to ART for the entire pregnancy, which may have important implications for the immediate and future health care needs of these children. In Malawi, maternal HIV exposure remains an important risk factor for infant morbidity and mortality,^43^ and there is evidence that HIV exposed, but uninfected infants have a higher risk of morbidity and mortality, perhaps because of a greater likelihood of immune dysfunction and/or low birth weight.^39,44,45^ Few adverse effects of in utero ART have been found in developed settings,^46–48^ but the short- and long-term effects of this exposure in populations where pregnancy nutrition is suboptimal and mothers are exposed to multiple infections are not well described.^30,49,50^ Longer follow-up with longitudinal studies of children exposed to ART are needed to understand better the longer-term sequelae of this early life exposure.

Incomplete data on HIV status and ART use is a limitation of our study. We found different patterns in fertility rates in women with unknown HIV status; however, as their status is unknown to the research team, this group will represent women who truly do not know their current HIV status (never tested or received results from a test) and those who know but were unwilling to disclose. In Malawi, opt-out HIV testing is routinely offered in antenatal care; hence, it is likely that women who have had a birth are more likely to know their status than women who have not. These factors make it difficult to interpret the fertility rates in women with unknown status. Exclusion of women who started ART during pregnancy further reduced our sample size in this category, making interpretation of fertility trends a challenge: for example, although there appeared to be an increase in fertility rates in young HIV-positive women on ART, the confidence limits were wide, which may have been because of a small number of women in this category. However, use of high-quality HDSS data means that we achieved high ascertainment of all live births and use of HIV test data from population-based HIV serosurveys, with high participation rates (73%–83%), will have minimized the potential for selection bias regarding HIV status. We do not capture information on ART care from clinics outside the HDSS area, so this could introduce bias if seeking care outside the area was associated with fertility. Inaccuracies in ART start and stop dates within clinic registers may have introduced some misclassification of women's exposure. We have assigned women to be HIV positive for 1 year before their first positive test because of the assumption that most people will have seroconverted long before they test. One year could be a conservative estimate and, with increasing access to HIV testing, it is likely that the length of time between seroconversion and testing positive will have changed over time. Without access to gestational age at birth, we have assumed each pregnancy to have lasted 9 months. This may have resulted in exclusion of some women who started ART before pregnancy but experienced a preterm birth, for which HIV infection and ART increase risk, thereby further attenuating fertility estimates in this group. Finally, although we attempted to control for factors known to be associated with HIV status, ART usage, and fertility (education, marital status, and age), some of these data were missing, and there may be other unmeasured factors confounding the observed associations.

## CONCLUSIONS

In rural Malawi, with widespread access to ART, despite decreasing overall fertility levels, fertility rates in HIV-positive women remain stable. Even without fertility levels in HIV-positive women matching those of HIV-negative women, the number of ART-exposed infants born has increased and is likely to continue to do so with continued implementation of the Option B+ mother-to-child transmission strategy. In this resource-constrained setting, determining and addressing the potential immediate and long-term health care needs of an increasing number of ART-exposed infants may be a challenge.
